# Soluble uric acid induces inflammation via TLR4/NLRP3 pathway in intestinal epithelial cells

**DOI:** 10.22038/ijbms.2020.44948.10482

**Published:** 2020-06

**Authors:** Chunling Ma, Xiaomin Yang, Qiulan Lv, Zhimei Yan, Zeqing Chen, Daxing Xu, Xiu Liu, Wan Yang, Shichao Xing

**Affiliations:** 1 Medical Research Center, the Affiliated Hospital of Qingdao University, Qingdao, Shandong Province, 266000, P. R. China; 2 Department of Obstctrics, the Affiliated Hospital of Qingdao University, Qingdao, Shandong Province, 266000, P. R. China; 3 Shandong Institute of Orthopaedics and Traumatology, the Affiliated Hospital of Qingdao University, Qingdao, Shandong Province, 266000, P. R. China; 4 School of Cardiovascular Medicine and Science, King’s College London, BHF Centre, London, SE5 9NU, United Kingdom

**Keywords:** Hyperuricemia, Inflammasome, Intestinal epithelium, Mechanism, ROS

## Abstract

**Objective(s):**

Hyperuricemia is a risk for cardiovascular and metabolic diseases, but the mechanism is ambiguous. Increased intestinal permeability is correlated with metabolic syndrome risk factors. Intestinal epithelial cells play a pivotal role in maintaining intestinal permeability. Uric acid is directly eliminated into intestinal lumen, however, the mechanism and effect of uric acid on intestinal epithelial cells is poorly explored. Here we carried out an analysis to identify the effect and mechanism of uric acid on intestinal epithelial cells.

**Materials and Methods:**

IEC-6 was exposed to different concentrations of uric acid to simulate the effect of uric acid on intestinal epithelial cells. Cell viability was determined by MTS assay. Protein content and mRNA were assessed using Western blotting and Q-PCR, respectively. Intracellular ROS was determined using flow-cytometry and fluorescence microscopy. Mitochondrial membrane potential was detected by immunofluorescence using a mitochondrial membrane potential assay kit with JC-1. Small interfering RNA transfection was used to suppress the expression of TLR4.

**Results:**

We found soluble uric acid alone increased the release of ROS, depolarized the mitochondrial membrane potential, up-regulated TSPO, increased the expression of TLR4 and NLRP3, and then activated NLRP3 inflammasome and NF-κB signaling, which further resulted in lower expression of tight junction protein and exerted adverse effects on intestinal epithelial cells. Furthermore, the elevated IL-1β could be restored by silencing of TLR4, indicating soluble uric acid induces inflammation via the TLR4/NLRP3 pathway.

**Conclusion:**

Soluble uric acid exerted detrimental effect on intestinal epithelial cells through the TLR4/NLRP3 pathway.

## Introduction

Growing evidence supports the hypothesis that hyperuricemia is an independent risk factor for hypertension, cardiovascular and metabolic diseases, but the mechanism is poorly understood. With research focusing on hyperuricemia and its complications, the pro-inflammatory effects of soluble uric acid (sUA) have been brought into attention (1-5). Studies showed that sUA could induce inflammation of vascular endothelial, renal proximal tubule epithelial, and hepatocytes cells, which were regarded as the key mechanism for explaining the metabolic syndrome induced by hyperuricemia (6, 7). However, few studies noticed the fact that uric acid is directly eliminated into intestinal lumen. Whether sUA induces intestinal epithelial cells (IECs) dysfunction and exerts adverse effects or not is still unknown.

IECs play fundamental roles in maintaining gut homeostasis and responding to pathogens by producing mucosal barriers, modulating host immune responses, and delivering bacterial antigens (8). The dysfunction of IECs can weaken epithelial barrier, and then promoting microbes and the metabolic products translocating into systemic circulation, which was favorable to drive systemic inflammation. Increased intestinal permeability is associated with the occurrence of atopic diseases, asthma, type1 diabetes and celiac disease (9-11). Kidney accounts for two-thirds of uric acid elimination, while one-thirds is excluded through gut, which indicates that intestinal epithelium is an important alternative way to uric acid secretion (12). In hyperuricemia patients, the intestine is under high levels of uric acid conditions, however, the effects of uric acid on IECs are less studied. Dysfunction of IECs induced by uric acid may be another crucial risk factor for onset of metabolic diseases in hyperuricemia. Studying the effect of uric acid on IECs may provide a new insight in explaining mechanism of hyperuricemia in metabolic syndromes and identify a novel therapeutic target.

Pattern recognition receptors (PRRs) including toll-like receptors (TLRs) and non-canonical nucleotide binding domain (NOD)-like receptor (NLRs) can recognize microbe associated molecular patterns (MAMPs) and initiate innate immune response(13). TLR4 and NLRP3 are the members of PRRs that are responsible for recognizing pathogen-associated molecular patterns (PAMPs) and activating cytokine signaling pathways. NLRP3 inflammasome is responsible for the maturation of pro-interleukin-1β (pro-IL-1β) and pro-interleukin-18 (pro-IL-18), thus initiating innate immune (14, 15). With research focusing on metabolic syndrome induced by hyperuricemia, growing evidence showed that soluble uric acid can also activate NLRP3 inflammasome in macrophages. However, whether soluble uric acid also functions as NLRP3 inflammasome activator on IECs remains unknown. It will be of great significance to decipher the effect and mechanism of uric acid on IECs. 

Thus, we presumed that soluble uric acid may directly damage IECs through the TLR4/NLRP3 pathway and then increase intestinal permeability, which promotes microbial metabolite translocation into systemic circulation, and then increase systemic inflammation. 

## Materials and Methods

***Cells culture and treatment***

Intestinal epithelial cells (IEC-6) purchased from ATCC (CRL-1592) were incubated in Dulbecco’s modified Eagle’s medium (DMEM) supplemented with 10% fetal bovine serum(FBS), 100 U/ml penicillin G, and 100 U/liter streptomycin in a humidified atmosphere containing 5% CO_2_ at 37 ^°^C. Cells were passaged every 2~3 days. In order to keep cells quiescent and minimize the influence of cell growth, FBS was reduced to 5% in all experiments. At 80% confluence, cells were exposed to different concentrations of uric acid for 24 or 48 hr. Uric acid concentration was chosen based on cell proliferation assay.

***Preparation of soluble uric acid***

Uric acid (UA) (Sigma, Saint Louis, USA) was dissolved in 1 M NaOH and filtered through a 0.22 μm syringe filter unit (Millipore), yielding a clear and faint yellow solution. The pH was adjusted within the normal range with 1 M HCl before use. Crystals were not detectable under a polarizing microscopy during cell incubation.

***Assay of cell viability***

To examine cytotoxic effects of sUA on IEC-6, cell viability was tested by 3-(4,5-dimethylthiazol-2-yl)-5-(3-carboxy-methoxyphenyl)-2-(4-sulfophenyl)-2H-tetrazolim (MTS) assays (Promega, USA). Briefly, IEC-6 cells were seeded in 96-well plates (0.25x10^5^ cells/well) and exposed to sUA (0, 2.5, 5, 10, 20, 40, and 50 mg/dl) for 24 and 48 hr, respectively. And then IEC-6 were cultured in serum free medium mixed with 20% MTS solution at 37 ^°^C for 2 hr. The absorbance was determined using a microplate reader at 490 nm. Cell viability was presented as percentage of the control group.

***Measurement of intracellular ROS and mitochondrial membrane potential (mt. ΔΨ)***

The level of intracellular ROS was measured using a Reactive Oxygen Species Assay Kit (DCFH-DA, Beyotime, Jiangshu, China). After stimulation with sUA, IEC-6 cells grown in 12-well plates were washed twice with PBS to remove the medium, and then incubated with 10 μM DCFH-DA in serum-free medium at 37 ^°^C for 30 min. After washing three times with PBS, the fluorescence was recorded on a flow-cytometer and fluorescent microscope.

Mitochondrial membrane potential kit with JC-1 (Beyotime, Jiangshu, China) was used to detect mt.ΔΨ. JC-1 enters the mitochondria in proportion to the membrane potential. Under a high mitochondrial membrane potential condition, JC-1 aggregates in mitochondrial matrix and forms J-aggregates, producing red fluorescence. However, under a low potential, JC-1 is monomer and produces green fluorescence. After stimulation with sUA, IEC-6 cells grown in 12-well plates were washed twice with PBS to remove the medium, and then incubated in the solution mixed with 50 µl JC-1, 8 ml ultrapure water and 2 ml 5X JC-1 buffer for 20 min at 37 ^°^C. Cells were washed three times with cold JC-1 buffer. The green and red fluorescence values were read on a multi well plate reader with excitation wavelengths at 488 nm and emission wavelengths at 527 nm (green emission) and 590 nm (red emission), respectively.

***Immunofluorescence and Western blot analysis of NF-kB p65 translocation***

After treatment with sUA (0, 2.5, 5, and 10 mg/dl) for 24 hr, cells were then fixed in 4% paraformaldehyde for 30 min, permeabilized in 0.5% Triton X-100 in PBS (pH 7.2) for 15 min, blocked in 5% goat serum at room temperature for 30 min, and incubated with antibodies against NF-κB p65 (CST, USA) at 4 ^°^C overnight. After washing with PBS, cells were incubated with fluorescently conjugated secondary antibodies for 1 hr at room temperature. Nuclei were visualized by staining with DAPI (Sigma). Fluorescence images were acquired using a fluorescent microscope. 

Total nuclear protein was extracted using nucleoprotein extraction kit (Beyotime, Jiangshu, China) and the content of NF-κB p65 was analyzed by Western blot.

***Small interfering RNA construction and transfection***

IEC-6 were transduced with either TLR4-specific lentiviral short hairpin RNA (shRNAs) or nonspecifc shRNA (shNS) using the lipofectamine 3000 transfection reagent (Invitrogen, USA) following manufacturer’s instructions. The knockdown of TLR4 gene was confirmed at protein level by Western blotting.

***RNA extraction and real-time quantitative PCR***

Total RNA was extracted by TRIzol reagent (TaKaRa, Tokyo, Japan) following the manufacturer’s instructions. The cDNA was synthesized by PrimeScript RT reagent Kit (Takara, Tokyo, Japan) according to the manufacturer’s instructions. Real-time PCR was performed in triplicate on an ABI 7500 Fast System using SDS software with SYBR® Premix Ex Taq™ Kit (Takara, Kyoto, Japan). The specific primers were designed using Primer 5 software and synthesized by Sangon Biotech. The specific primers were designed using Primer 5 software and synthesized by Sangon Biotech. The sequences of the primers used were 5’-GGACTCTGCCCTGCCACCATTTA-3’ (forward) and 5’-CTTGTGCCCTGTGAGGTCGTTGA-3’ (reverse) (for TLR4 gene); 5’-AGAAGCTGGGGTTGG TGAATT-3’ (forward) and 5’-GTTGTCTAACTCCAGCATCTG-3’ (reverse) (for NLRP3 gene); 5’-CACCTCTCAAGCAGAGCACAG-3’ (forward), and 5’-GGGTTCCATGGTGAAGTCAAC-3’ (reverse) (for IL-1β gene); 5’-TACACTGGTCAGCTGGCTCTGAA-3’ (forward), and 5’-ACAAGC ATGAGGTCCACCAAA G-3’ (reverse) (for TSPO gene); 5’-GCCAAA AGG GTCATCATCTCCG-3’ (forward), and 5’-ACATTGGGGGTAGGAACACGGA-3’ (reverse) (for GAPDH gene). The fold increase of mRNA abundance was calculated with 2^−ΔΔCt ^method and the GAPDH gene was used as the reference. 

***Western blot analysis***

After stimulation with sUA for 24 hr, the cells were rinsed with PBS twice and lysed in radioimmuno-precipitation assay (RIPA) buffer containing 1% PMSF on ice for 15 min. After scraping and pelleting, the cell lysate was centrifuged at 15,000 rpm for 15 min with the supernatant collected. The supernatant was mixed with SDS-PAGE loading buffer and immediately heated at 100 °C for 5 min. Samples with equal amount of protein were subjected to electrophoresis on 10% or 12% sodium dodecyl sulfate polyacrylamide gel electrophoresis (SDS-PAGE) and then transferred onto polyvinylidene fluoride (PVDF) membrane (Millipore, Billerica, MA, USA). After blocked at room temperature for 2 hr with 5% skimmed milk, the membrane was incubated at 4 ^°^C overnight with the following respective primary antibodies, mouse against TLR4 (1:1000, SantaCruz, MA, USA), rabbit against NLR3 (1:1000, Abcam, MA, USA), rabbit against p65 (1:1000, CST, USA), rabbit against H3 (1:1000, CST, USA), rabbit against IL-1β (1:500, Abcam, USA), and rabbit against IL-6R (1:500, Sino Biological, China). After washing with PBST and incubation with horseradish peroxidase-conjugated secondary antibody (1:3000, Abcam, MA, USA) at 37 ^°^C for 1 hr, the protein signal was detected on an imaging system by using a chemiluminescence kit (Millipore, Billerica, MA, USA). Protein bands were analyzed by Image-Pro Plus software and normalized to the synthesis of β-actin or H3.

***Measurement of culture supernatant IL-1β***

Concentrations of IL-1β in culture supernatant was measured using ELISA Kit (mibio, China) according to the manufacturer’s instructions in duplicate. Absorbances at 405 nm were measured by a microplate reader.

***Statistical analysis***

All values were presented as mean±SEM. Statistical analysis was performed by one-way analysis of variance (ANOVA) followed by Duncan’s multiple range t-test using SPSS 17.0. A *P*<0.05 was believed to be statistically signiﬁcant.

## Results

***Soluble uric acid involved in IEC-6 cytotoxicity***

**Figure 1:**
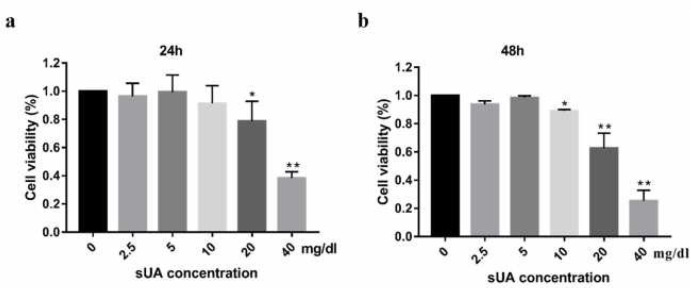
Effects of soluble uric acid on IEC-6 viability

To explore cytotoxicity effect of uric acid on IEC, we evaluated cell proliferation in the presence of uric acid (0, 2.5, 5, 10, 20, and 40 mg/dl) at 24 and 48 hr, respectively. Figures 1A and B showed that when uric acid was present within a high concentration range (20–40 mg/dl), cell proliferation was decreased significantly, and the effect was time and concentration-dependent. However, no significant inhibition was observed when uric acid was lower than 10 mg/dl. In order to avoid inhibiting cell proliferation, all subsequent experiments were performed using 2.5, 5, and 10 mg/dl uric acid for 24 hr. 

***Soluble uric acid resulted in mitochondrial dysfunction***

**Figure 2:**
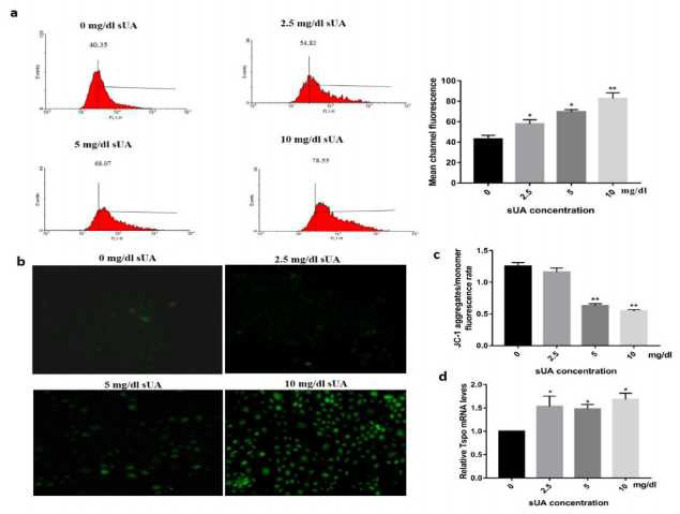
Soluble uric acid dysfunction mitochondria

To explore whether there is an oxidative stress response induced by sUA, we detected ROS generation by measuring oxidized DCFH-DA using flow-cytometry and a fluorescence microscope. As demonstrated in Figures 2A and B, overproduction of ROS was observed even at low concentrations of sUA (2.5 mg/dl). High concentration of sUA (10 mg/dl) dramatically increased ROS generation (*P*<0.01). We further explored whether the elevated ROS production was due to mitochondrial dysfunction induced by uric acid. As illustrated in Figure 2C, high concentration of sUA markedly depolarized mt. ΔΨ, which was highly correlated with the production of ROS. 

Given the observed change in mt. ΔΨ, we next analyzed the potential gene responsible for ROS production. We detected the expression of Tspo. As shown in Figure 2D, coincident with the change of mt. ΔΨ, sUA up-regulated Tspo expression.

***Soluble uric acid activated NLRP3 inflammasome***

**Figure 3:**
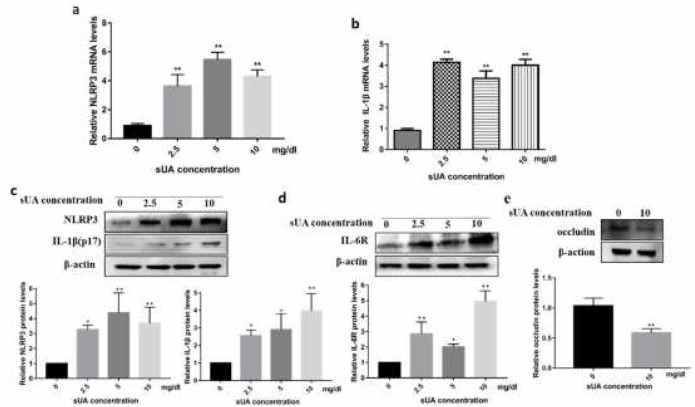
Soluble uric acid activate NLRP3 inflammasome

We observed elevated expression of NLRP3 both at mRNA and protein levels as well as mature IL-1β when treated with 10 mg/dl sUA (Figures 3A, B, and C). However, we failed to detect IL-1β in the culture supernatants. Of particular note, we observed increased IL-6R expression in cell lysate in tandem with IL-1β (Figure 3D). Meanwhile, occludin expression was markedly lower when treated with 10 mg/dl sUA (Figure 3E). 

***Soluble uric acid induced inflammation was dependent on TLR4***

**Figure 4:**
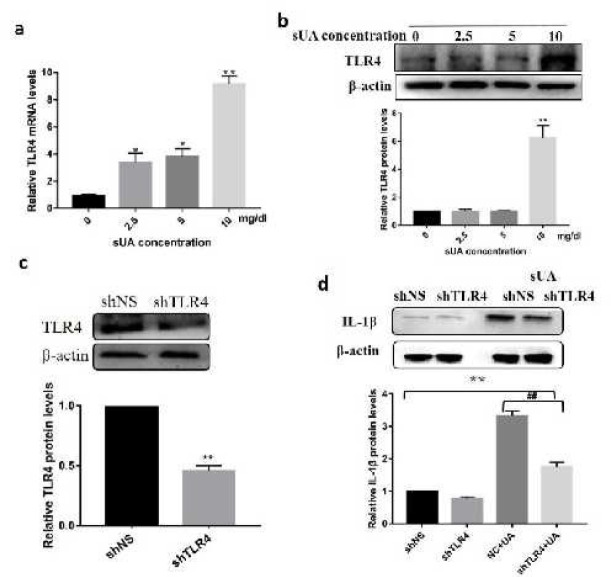
Soluble uric acid induced inflammation through TLR4 signaling pathway

We further investigated whether TLR4 was involved in sUA induced inflammation. We found sUA up-regulated TLR4 expression both at mRNA and protein levels (Figures 4A and B). To further directly assess the roles of TLR4 in sUA induced inflammation, we silenced TLR4 expression using lentiviral short hairpin RNA (shRNA) against TLR4 (shTLR4) (Figure 4C). Lower expression of TLR4 resulted in a dramatic reduction of mature IL-1β as demonstrated in Figure 4D. 

***Soluble uric acid activated NF-κB signaling pathway***

**Figure 5:**
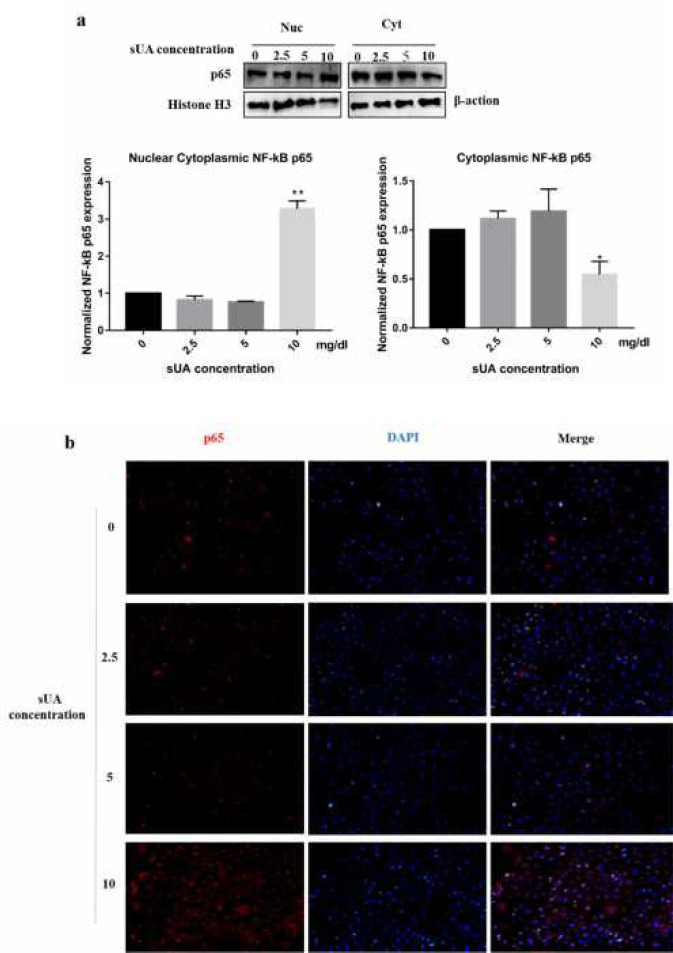
NF-κB signaling involved in sUA induced inflammation

We next sought to determine whether NF-κB signaling was activated in uric acid induced inflammation. The activation of NF-κB was evidenced by translocation of NF-κB into the nucleus. sUA (10 mg/dl) markedly increased NF-κB p65 nuclear translocation, while no signal was detected at low concentration (Figures 5A and B). 

## Discussion

Evidence increasingly suggests that hyperuricemia is associated with metabolic syndrome such as kidney disease, hypertension, cardiovascular, and atherosclerosis, but the mechanism is unknown. Intestinal epithelial cells play vital roles in preventing luminal damaging agent transfer into systemic circulation, which contributes to the progression of metabolic syndrome (16). Revealing the effects and underlying mechanisms of uric acid on intestinal epithelial cells is significant to explain the metabolic syndrome induced by hyperuricemia. Research has demonstrated that sUA can induce vascular endothelial cell dysfunction and renal mesangial fibrosis. However, the effects of sUA on intestinal epithelial cells are little studied. In this study, we found that uric acid directly damaged intestinal epithelial cells. The inflammation induced by uric acid resulted in decreased expression of occludin. 

MSU induced inflammation has been widely studied, but the effects of sUA on intestinal epithelial cells is ambiguous. Oxidative stress, which plays a crucial role in regulating inflammation, host immune response and apoptosis, is essential for inducing intestinal barrier damage. The overproduction of ROS induced by uric acid was also observed in previous studies (17). Similarly, we demonstrated that uric acid induces ROS overproduction in tandem with depolarization of mt.ΔΨ. However, it is interesting to note that intestinal epithelial cells are more sensitive to oxidative stress response induced by uric acid. ROS is the key factor for activating NLRP3 inflammasome, thus intriguing a series of inflammations. The NLRP3 inflammasome is response to pathogen-associated and damage-associated molecular patterns. Once the NLRP3 inflammasome is activated, it can result in the release of IL-1β, and then amplifying inflammation. Dysregulated activation of NLRP3 inflammasome has been shown to be associated with insulin resistance, non-alcoholic fatty liver disease, cardiovascular complications, atherosclerosis and type 2 diabetes (18-20). Increasing data strongly indicate uric acid can also activate NLRP3 inflammasome (21, 22). As previously reported, we also showed that uric acid seems to have a similar role in intestinal epithelial cells. We showed uric acid activated NLRP3 inflammasome in intestinal epithelial cells, and then increased mature IL-1β expression, which resulted in disruption of tight junctions and increased intestinal permeability (23). Whereas, a unifying trigger for inflammasome activation has not yet been firmly established. Increasing evidence strongly suggests that mitochondrial dysfunction is a crucial factor for NLRP3 inflammasome activation (24). In this study, we observed the depolarization of mt.ΔΨ and up-regulation of Tspo, which is the sign of mitochondrial dysfunction. Tspo is a protein that interferes with redox balance. Binding with voltage-dependent anion channel (VDAC), it can reduce mitochondrial coupling, promoting ROS overproduction and resulting in the accumulation of dysfunctional mitochondria (25, 26). It is noteworthy that Tspo is responsible for ROS overproduction, which results in the depolarization of mt.ΔΨ, and then further inducing K^+^ efflux . While K^+^ efflux is a pre-requisite for NLRP3 inflammasome activation and IL-1β maturation. Thus, it is possible that sUA may activate NLRP3 inflammasome through up-regulating Tspo, and then igniting inflammation response, which results in intestinal epithelial cell dysfunction. However, the hypothesis needs more empirical evidences.

Researchers found that sUA induces epithelial cell damage in a TLR4-dependent manner. TLR agonists is a strong stimulus for NLRP3 inflammasome activation (27). TLR4 has been recognized as an sUA receptor and plays a pivotal role in amplifying inflammatory effects. In the present study, we also found sUA up-regulated TLR4 expression and suppressing TLR4 decreased IL-1β expression. These indicated that TLR4 is responsible for the activation of inflammasome induced by sUA in IEC-6. Furthermore, sUA may have direct pro-inflammatory effects through activation of the NF-κB pathway. The activation of NF-κB increases expression of pro-inflammatory cytokines including TNF-α, IL-1β, IL-6 and enzymes that produce ROS, which in turn, further activate NF-κB. In our study, we demonstrated that NF-κB involved in uric acid induced intestinal cell dysfunction. NF-κB activation was observed only at high concentration, suggesting that multiple signal pathways may be involved in sUA induced IL-1β production, such as mitogen-activated protein kinase (MAPK), protein Kinase C (PKC). 

Only about one-thirds of uric acid is excreted through the intestines, and some of them are even fermented by microflora. However, hyperuricemia is usually associated with renal impairment, which results in uric acid secretion through the intestines for replenishment, leading to intestinal microenvironment disorders (28). Increased intestinal permeability could drive translocation of bacterial components, fueling systemic inflammation and impairing cellular antibacterial function, which is a strong risk factor for metabolic syndrome (29, 30). Although flora disturbance may be another factor for inducing intestinal barrier dysfunction, the directly damaging effect of uric acid on intestinal epithelial cells cannot be ignored. Intestinal epithelial cell damage may represent a potential link for understanding the correlation between hyperuricemia and metabolic syndrome. Although further studies are needed to verify, we proposed that future therapeutic strategies for metabolic diseases should pay more attention to the intestines.

After treatment with different concentrations of sUA for 24 or 48 hr, cell viability was determined by MTS assay. (a) Viability of IEC-6 after treatment with sUA for 24 hr. (b) Viability of IEC-6 after treatment with sUA for 48 hr. Cell viability analysis was normalized to the control group. Values are means ±SEM of three separate experiments. *P<0.05 and *P<0.01 vs control

sUA: Soluble uric acid

(a-b) IEC-6 were treated with uric acid (0, 2.5, 5, 10 mg/dl) for 24 hr, and then stained with DCFH-DA and analyzed by flow-cytometry (a) and fluorescence microscope (b). (c) Mitochondrial ΔΨ was detected using mitochondrial membrane potential kit. Ratio of J-aggregate to JC-1 monomer represents mitochondrial ΔΨ. (d) Tspo was analyzed by Q-PCR. Data are presented as the means ±SEM obtained from 3 different experiments. *P<0.05 and **P<0.01 vs control

mitochondrial ΔΨ: mitochondrial membrancne potential

IEC-6 was treated with sUA (0, 2.5, 5, and 10 mg/dl) for 24 hr. (a-b) NLRP3 and IL-1β mRNA levels were analyzed by Q-PCR. (c-e) Cell extracts were analyzed by Western blotting for NLRP3, IL-1β, IL-6R, and occludin. Data are representative of three independent experiments and are presented as means±SEM. *P<0.05 and **P<0.01 vs control

sUA:soluble uric acid

(a-b) After treatment with different concentrations of sUA for 24 hr, TLR4 expression was analyzed by Q-PCR and Western blotting. IEC-6 cells were transfected with either nonsilencing shRNA or shRNA directed against TLR4 as indicated. (c-d) 24 hr later, cells were primed with uric acid (10 mg/dl) for 24 hr. Cell lysates were analyzed by Western blotting for TLR4 and IL-1β. Data are representative of three independent experiments and presented as means±SEM. *P<0.05 and **P<0.01 vs control (a and b) or shNS (c and d), ##*P<*0.01 vs shNS+sUA group

shRNAs: short hairpin RNA, shNS: nonspecific short hairpin, RNA,shTLR4,TLR4-specific lentiviral short hairpin RNA

(a) Cells were treated with increasing concentrations of sUA for 24 hr. Cytoplasmic and nucleus protein were analyzed by Western blotting for NF-κB p65 activation. (b) After primed with uric acid for 24 hr, cells were fixed, and NF-κB p65 translocation was evidenced by immunofluorescence. Nuclei were stained with DAPI (blue). Data are representative of three independent experiments and are presented as means ±SEM. *P<0.05 and **P<0.01 vs control

Nuc, nuclei; Cyt, cytoplasmic, sUA: soluble uric acid

## Conclusion

Our results showed that uric acid induces inflammation and exerts adverse effects on intestinal epithelial cells through the TLR4/NLRP3 pathway, inducing mitochondrial dysfunction and triggering NF-κB signaling, which resulted in decreased expression of tight junction protein. The elevated IL-1β could be restored by silencing of TLR4. Our study represents a new insight to illuminate the mechanism of hyperuricemia with metabolic syndrome and provide a novel therapeutic target.
